# Economic impact of extending reflexed cryptococcal antigenaemia CD4 threshold in South Africa

**DOI:** 10.4102/sajhivmed.v25i1.1621

**Published:** 2024-10-03

**Authors:** Naseem Cassim, Lindi-Marie Coetzee, Manuel P. da Silva, Deborah K. Glencross, Wendy S. Stevens

**Affiliations:** 1 Wits Diagnostic Innovation Hub, Faculty of Health Sciences, University of the Witwatersrand, Johannesburg, South Africa; 2 National Priority Programme, National Health Laboratory Service, Johannesburg, South Africa; 3 Faculty of Health Sciences, University of the Witwatersrand, Johannesburg, South Africa

**Keywords:** HIV, cryptococcal disease, reflexed, cryptococcal antigenaemia, cost

## Abstract

**Background:**

Reflexed cryptococcal antigenaemia (CrAg) testing has been offered since 2016 in South Africa, on remnant CD4 specimens, for people with a count < 100 cells/μL. Local guidelines recommended extending testing to 200 cells/μL.

**Objectives:**

This study assessed the cost per result and annual equivalent costs (AEC) for CD4 counts < 100 cells/μL and 100 to 200 cells/μL, as well as determining the cost to find one CrAg-positive case.

**Method:**

An ingredients-based costing was used to determine the cost per result. The CrAg detection rate for < 100 cells/μL was obtained from operational reports of 2019. For 100 cells/μL to 200 cells/μL, a CrAg detection rate of 2% was assumed. One-way sensitivity analysis determined the impact of varying CrAg detection rates on the cost to find one case. Local data from the Western Cape province, which offers testing for counts of 100 cells/μL to 200 cells/μL, from September 2022 to January 2023, were interrogated to establish detection rates.

**Results:**

There were 283 240 (AEC: $1 670 370) specimens with counts of < 100 cells/μL and 300 624 (AEC: $1 772 890) with counts of 100 cells/μL to 200 cells/μL. A cost per result of $5.897 was reported. The cost to find one CrAg case ranged from $589.74 to $73.72 for a detection rate of 1% to 8%. Local data for a count of 100 cells/μL to 200 cells/μL revealed a CrAg detection rate of 1.6%.

**Conclusion:**

The study findings reveal that extending reflexed CrAg testing to 200 cells/μL would double test volumes with fewer positive cases reported for those with a count of 100 cells/μL to 200 cells/μL.

**What this study adds:** This study provides insights into the diagnostic costs of extending reflexed cryptococcal antigenaemia (CrAg) screening from a CD4 count < 100 cells/μL to a threshold of < 200 cells/μL. The study findings show that including all CD4 < 200 cells/μL for CrAg reflexed testing would double the diagnostic cost while finding fewer positive cases for treatment.

## Introduction

The National Health Laboratory Service (NHLS) is the largest diagnostic pathology service in South Africa, with the responsibility of supporting the national and provincial health departments in the delivery of healthcare.^1^ This is achieved through a national network of laboratories that provides access to diagnostic services to more than 80% of the population.^1^ The NHLS performs the majority of HIV, tuberculosis, and cervical cancer testing for the public health sector.^1^

The 2019 national HIV guidelines recommended that people living with HIV (PLHIV) are eligible to start antiretroviral therapy (ART) regardless of age, CD4 count, and clinical stage.^2^ A CD4 count remains a valuable indicator of the immune status of PLHIV. Initially, CD4 counts were used to stratify HIV disease risk, identify patients eligible for ART, and monitor treatment failure.^3^ Since the inception of the ‘universal test and treat (UTT)’ guidelines by the World Health Organization (WHO) in 2016, ART initiation is not contingent on a CD4 count.^4^ Local 2019 guidelines, however, recommend CD4 testing preceding ART initiation to identify eligibility for cotrimoxazole preventive therapy (CPT), defer ART for patients with drug-sensitive tuberculosis and to assess immune status at 12 months on ART.^4^ After 12 months, it is also recommended that CD4 testing should be repeated every 6 months until the patient meets the criteria to discontinue CPT and/or the HIV viral load is below 1000 copies/mL.^2^ Similar recommendations were provided in the 2023 guideline updates.^5^

Guidelines for cryptococcal meningitis were first released in South Africa in 2007 by the Southern African HIV Clinicians Society (SAHCS), who recommended that patients with symptoms of meningitis should be screened.^6^ At the time, a lumbar puncture was essential for the confirmatory diagnosis of suspected meningitis.^6^ The culture of *Cryptococcus* species is considered as the gold standard for diagnosis and may take up to 14 days for confirmation.^6^ Therefore, the India ink test was recommended for patient screening, followed by cryptococcal antigenaemia (CrAg) testing.^6^

Due to difficulties in performing lumbar puncture, there was a need to migrate to antigen-based screening on blood to predict cryptococcal meningitis (CM). The antigen could either be requested by the healthcare worker when a CD4 count < 100 cells/μL was reported (provider-initiated) or as a reflexed test in the laboratory subsequent to confirmed CD4 (latter using a rule-based trigger in the laboratory information system).

Two studies were conducted to assess these screening approaches. A pilot study to offer CrAg laboratory-based reflexed testing was initiated at ~500 health facilities in the Gauteng and the Free State provinces.^7^ NHLS CD4 laboratories performed a CrAg reflexed test on a remnant CD4 specimen with a confirmed count < 100 cells/μL using the IMMY (Immy Mycologics, Norman, Oklahoma, United States) lateral flow assay (LFA).^7^ The Western Cape province adopted provider-initiated CrAg screening in 2012. They reported that only 26.6% of eligible patients were screened and unscreened patients were nearly twice as likely to develop cryptococcal disease.^8^

These studies generated important insights into operational challenges for a national screening programme.^7,8^ A cost-effectiveness analysis was conducted to compare these screening strategies.^7,8^ The reflexed screening strategy was based on the pilot study, while provider-initiated screening entailed the healthcare worker ordering a CrAg test during a consultation with a patient with either an available CD4 count or symptoms of CM.^9^ The reflexed CrAg screening strategy was more likely to be cost saving, or have low additional costs per additional year of life saved, when compared to provider-initiated screening.^9^

Based on this evidence, a national reflexed CrAg testing programme was introduced in 2016 for PLHIV with a CD4 count < 100 cells/μL.^8,9,10^ Subsequently, the National Department of Health HIV guidelines were updated to include reflexed CrAg testing.^2,3^ The current South African HIV guidelines recommend that a reflexed CrAg test should be done automatically by the laboratory on all CD4 counts < 100 cells/μL.^5^ PLHIV with a CrAg-positive test in the absence of symptoms or signs of meningitis, and if the lumbar puncture is negative for CM, can be initiated on ART.^5^ Those with confirmed CM should defer ART for 4 weeks to 6 weeks until antifungal treatment has been completed.^5^

Updated WHO guidelines of 2021 were consistent with earlier CrAg testing recommendations of the WHO since 2011.^2,11,12,13^ However, local SAHCS guidelines recommended that reflexed CrAg testing should be extended to include specimens with a CD4 count between 100 cells/μL and 200 cells/μL.^10^ A number of studies reported that CrAg screening for PLHIV with a CD4 < 100 cells/μL would potentially miss cases of CM in patients with a CD4 between 100 cells/μL and 200 cells/μL.^10,14,15^ Furthermore, it was reported that offering reflexed CrAg screening for a count between 100 cells/μL and 200 cells/μL would provide an important mortality benefit.^10^ There is limited local data on the cost of extending reflexed CrAg screening to a CD4 threshold of 200 cells/μL.

### Aim

The objectives of this study were to assess the economic impact of extending reflexed CrAg testing to a CD4 threshold of 200 cells/μL in South Africa. Furthermore, the study aimed to assess the cost per result, annual equivalent costs (AEC), and the cost to find one CrAg-positive patient for a count of < 100 cells/μL and 100 cells/μL to 200 cells/μL.

## Research methods

### Context

Data are reported for reflexed CrAg testing performed across 47 CD4 laboratories within the NHLS for the 2019 calendar year.^1^ The CD4 laboratories are distributed across all nine provinces and use a national laboratory information system rule-based algorithm to identify specimens that require reflexed CrAg testing. CD4 testing was offered using the FC500 MPL/CellMek and Aquios CL cytometers. These platforms were supplied by Beckman Coulter (Beckman Coulter, Inc., Miami, Florida, United States) as per the national tender agreements.^16,17^ Irrespective of instrument used, all laboratories utilised the same CD4 PanLeucoGating (PLG) reagents and national standard operating procedures.^16^ CrAg testing was done at the CD4 testing laboratories, using the LFA provided by IMMY (Immy Mycologics, Norman, Oklahoma, United States) as per a national tender agreement.^9,10^

### Study design

A cross-sectional study design was used to assess the economic impact of extending reflexed CrAg screening to a CD4 threshold of 200 cells/μL.

### Costing analysis

The accounting stance was as the provider of testing. An exchange rate of R14.45/$1 was assumed based on the 2019 period average reported by the International Monetary Fund.^18^ An error rate of 1% and an annual discount of 4% were applied (overheads were excluded). The costing analysis determined the cost per result and AEC for reflexed testing performed at the Tambo Memorial CD4 laboratory. The ingredients-based costing analysis included laboratory equipment, reagents and staff. For laboratory equipment, the costs for a pipette and specimen racks were included. Reagents consisted of the CrAg IMMY LFA assay (Immy Mycologics, Norman, Oklahoma, United States) and associated test consumables. For staff costs, the NHLS cost-to-company salary scales for medical technologists were used. Working days per annum were calculated considering public holidays (*n* = 12), Sundays (testing not performed), and annual/sick leave. The nett working minutes per year were used to calculate the cost per minute. The staff cost to perform one test was multiplied by annual test volumes to determine the AEC. Reagent prices were obtained from supplier quotations. The costs reported here are for laboratory testing and exclude patient management. The consolidated health economic evaluation reporting standards were used for this costing analysis.^19^

### Data analysis

Aggregate CD4 test volumes by result category (< 100 cells/μL, 100 cells/μL – 200 cells/μL, > 200 cells/μL) were calculated. The CrAg detection rate for a count < 100 cells/μL was determined. For a count between 100 cells/μL and 200 cells/μL, a CrAg detection rate of 2% was assumed based on local and published data.^14,20,21^ Based on the costing analysis, the national AEC for reflexed CrAg testing for a count < 100 cells/μL, and 100 cells/μL to 200 cells/μL, was extrapolated. A one-way sensitivity analysis was conducted to assess the cost to find one case based on varying CrAg detection rates (1% – 8%) for a count of 100 cells/μL to 200 cells/μL. The Western Cape province recently commenced offering CrAg testing for a count of 100 cells/μL to 200 cells/μL. These data were analysed to assess the detection rate for the period between September 2022 and January 2023 to determine the cost of finding a single positive case. The average cost to find one CrAg case was also determined.

### Ethical considerations

Ethical clearance was obtained from the Human Research Ethics Committee (HREC) (Medical) at the University of the Witwatersrand (M220163). Specimen-level data were extracted without any patient identifiers. Patient consent was not required as secondary laboratory data were used.

## Results

Data are reported for 2 875 719 CD4 tests, of which 283 240 (9.8%) reported a count of < 100 cells/μL, and 300 624 (10.5%) a count of 100 cells/μL to 200 cells/μL. For 2019, the CrAg detection rate for reflexed testing was 6.2% nationally (ranging from 5.5% to 7.5% by month).

### Costing analysis

A cost per result of $5.897 was reported for reflexed CrAg testing for a count < 100 cells/μL at the Tambo Memorial laboratory (Table 1). This was based on annual CrAg test volumes of 283 240 for 2019. Due to the similar national test volumes for a count of < 100 cells/μL and 100 cells/μL to 200 cells/μL, the same cost per result was used.

**TABLE 1:** Assessing the cost of reflexed cryptococcal antigenaemia testing for a CD4 < 100 cells/μL performed at the Tambo Memorial laboratory in South Africa for the 2019 calendar year.

Cost category	Cost per result (USD)	Contribution (%)
Reagents	4.381	74.3
Laboratory equipment	0.004	0.1
Staff	1.512	25.6

**Total**	**5.897**	**100.0**

Note: The cost per result is reported.

USD, United States Dollar.

### National cost analysis

An AEC of $1 670 370 (< 100 cells/μL) and $1 772 890 (100 cells/μL – 200 cells/μL) was calculated. Based on the CrAg detection rates of 7% (< 100 cells/μL) and 2% (100 cells/μL – 200 cells/μL), the cost to find one case was $84.25 for < 100 cells/μL and $294.87 for 100 cells/μL to 200 cells/μL (Table 2).

**TABLE 2:** Determining the annual equivalent costs for offering reflexed cryptococcal antigenaemia testing for a CD4 count < 100 cells/μL and 100 cells/μL to 200 cells/μL.

CD4 category (cells/μL)	Annual test volumes		Cost per result (USD)	Annual equivalent cost (USD)	CrAg detection rate (%)	CrAg-positive specimens	Cost to find one CrAg-positive case (USD)
	*n*	%					
< 100	283 240	48.5	5.897	1 670 370	7.0	19 827	84.25
100–200	300 624	51.5	5.897	1 772 890	2.0	6012	294.87

**Total**	**583 864**	**100.0**	**5.897**	**3 443 259**	-	-	-

Note: The analysis was conducted for national testing performed across 47 CD4 laboratories in South Africa. The cost per result was determined at the Tambo Memorial laboratory. The CrAg detection rate was used to determine the cost to find a single CrAg-positive case.

CrAg, cryptococcal antigenaemia; USD, United States Dollar.

### One-way sensitivity analysis

**FIGURE 1:**
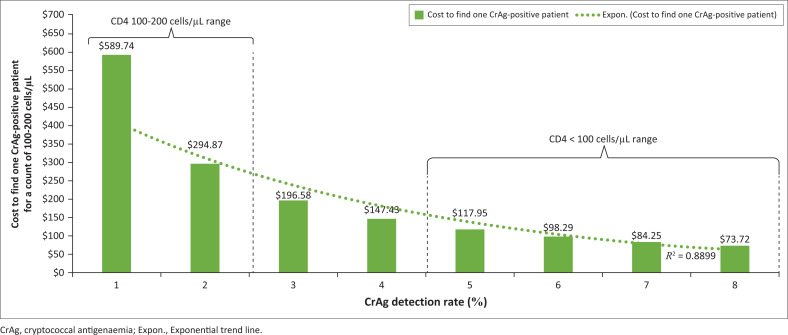
One-way sensitivity analysis to assess the impact of varying cryptococcal antigenaemia (CrAg) detection rates from 1% to 8%, for a CD4 between 100 cells/μL and 200 cells/μL, on the cost to find one CrAg-positive patient. The cost per result was assumed to be fixed at $5.897. A CrAg positivity of ≤ 2% was assumed for a CD4 100 cells/μL to 200 cells/μL compared to between 5% and 8% for a count < 100 cells/μL. The exponential trend line was reported.

The cost to find one CrAg case ranged from $589.74 to $73.72 for a detection rate of 1% to 8%. For a CD4 of < 100 cells/μL, the cost to find one CrAg case with a detection rate of 5% was $117.95, $98.29 for 6%, and $84.25 for 7%. A detection rate of 1% to 3% would be between 7- and 2.3-fold more expensive to find a case when compared to 7% (Figure 1). For the exponential trend line, an *R*^2^ value of 0.8899 was reported, which confirms the decreasing cost to find one CrAg case with increasing detection rates.

### Western Cape analysis (100 cells/μL to 200 cells/μL)

For the period September 2022 to January 2023, the percentage of specimens with a count < 100 cells/μL was 15.1% of total volumes, and 100 cells/μL to 200 cells/μL, 16.2%. CrAg test volumes for CD4 100 cells/μL to 200 cells/μL performed in the Western Cape province ranged from 1557 (December 2022) to 2190 (January 2022). The overall CrAg detection rate was 1.6%, ranging from 1.1% (October 2022) to 2.1% (December 2022). In this setting, the cost to find one case ranged from $278.25 to $525.36 (Table 3). Across this period, the average cost to find one case was $379.40.

**TABLE 3:** Analysis of the cryptococcal antigenaemia (CrAg) detection rate in the Western Cape province for a CD4 count of 100 cells/μL to 200 cells/μL performed between September 2022 and January 2023.

Month	Year	Test volumes		CrAg-positive specimens (*n*)	CrAg detection rate (%)	Cost per result† (USD)	Annual equivalent cost (USD)	Cost to find one CrAg-positive case (USD)
		*n*	%					
September	2022	2149	21.0	31	1.4	5.897	12 673.44	408.82
October	2022	2138	20.9	24	1.1	5.897	12 608.57	525.36
November	2022	2189	21.4	37	1.7	5.897	12 909.33	348.90
December	2022	1557	15.2	33	2.1	5.897	9182.20	278.25
January	2023	2196	21.5	34	1.5	5.897	12 950.61	380.90

**Total**	**-**	**10 229**	**100.0**	**159**	**1.6**	**5.897**	**60 324.15**	**-**

**Average**	**-**	**-**	**-**	**-**	**-**	**-**	**-**	**379.40**

Note: The cost per result was based on the one-way sensitivity analysis for varying detection rates ranging from 1% to 8%. The CrAg detection rate was used to determine the cost to find a single CrAg-positive case.

CrAg, cryptococcal antigenaemia; USD, United States Dollar.

† , Cost per result is based on the detection rate with values obtained from the one-way sensitivity analysis.

## Discussion

Extending reflexed CrAg testing to a CD4 threshold of 200 cells/μL would effectively double the AEC for the national programme. The study confirmed previous work that showed a higher CrAg detection rate in PLHIV with a CD4 count < 100 cells/μL when compared to the 100 cells/μL to 200 cells/μL group.^14^ The cost to find one CrAg case was 3.5-fold more expensive for a count of 100 cells/μL to 200 cells/μL compared to < 100 cells/μL. This implies that while AEC would double for extending reflexed CrAg testing to a CD4 threshold of 200 cells/μL, the actual cost to find a positive CrAg case would be higher due to lower detection rates. Furthermore, for the same AEC, far fewer patients would be identified who are eligible for antifungal treatment. However, finding these patients timeously and putting them onto treatment could result in CM reduction.

The Western Cape data reported a CrAg detection rate of between 1.1% and 2.1% for a count between 100 cells/μL and 200 cells/μL. The one-way sensitivity analysis revealed that higher detection rates decreased the cost to find one CrAg-positive case, with an 8-fold change from 1% to 8%. Significantly higher costs per positive CrAg outcome were reported for a CrAg detection rate of 1% to 4% when compared to > 5%, without affecting AEC. There is an almost logarithmic increase in the cost to find one CrAg-positive case with decreasing detection rates.

These findings indicate that a national prevalence study would be of value to better determine the CrAg detection rate for a count of 100 cells/μL to 200 cells/μL. A local unpublished survey reported that the overall CrAg prevalence for all patients with a CD4 count of < 200 cells/μL was 4.6%, compared to 6.8% for < 100 cells/μL and 2.4% for 100 cells/μL to 200 cells/μL.^20^

A systematic review of 60 studies reported that the pooled prevalence of CrAg was 6.5% (95% confidence interval [CI]: 5.7% – 7.3%) for a CD4 count of ≤ 100 cells/μL versus 2.0% (95% CI: 1.2% – 2.7%) for a count of 101 cells/μL to 200 cells/μL.^14^ This is similar to our study findings which showed that CrAg detection rates were higher among those with a lower CD4 cell count.^14^ In addition, the data reported in the Western Cape confirm the pooled prevalence for a count of 101 cells/μL to 200 cells/μL reported by Ford et al.^22^ Another local study reported a CrAg prevalence of 1.9% for a count of 100 cells/μL to 200 cells/μL, in accordance with our findings.^23^

As the study did not conduct a cost-effectiveness analysis, it would be difficult to assess whether extending CrAg screening to a CD4 threshold of 200 cells/μL would result in the reduction of disability-adjusted life years saved. The post-diagnostic costs associated with treatment have not been factored into this study. These would include pre-emptive treatment to avoid hospitalisation, hospital costs, and post-hospital costs.^9^ A cost-effectiveness analysis would include both screening and treatment costs. However, given the limited public health resources in a country with a high burden of both communicable and non-communicable diseases, cost-effectiveness analysis should guide policy choices.^24^

Extending reflexed testing to a CD4 threshold of 200 cells/μL would also double the current workload, requiring careful planning as additional staff may need to be employed for both the pre-analytical and analytical phases within the laboratory. The current LFA assay is a manual test method with staff contributing over one-quarter of the cost per result. Doubling reflexed CrAg test volumes may require the need to migrate testing to a more automated platform, such as enzyme-linked immunosorbent assay (ELISA).^25^ Some of the challenges with using ELISA plates is that the cost per result would be lowest with all wells being tested in a single run. When reflexed in some of the smaller laboratories, it would take some time to fill up an ELISA plate, which may indirectly increase turnaround time and diagnostic costs. The current batch size for LFA testing is 30 specimens. In smaller laboratories, obtaining a batch of 96 specimens for ELISA testing may delay diagnosis. Supply chain management, staffing, platform choice, and workflow considerations must be carefully deliberated, should test volumes double.

### Limitations

A limitation of this study is that only specimen-level data for reflexed CrAg testing were used, which excluded provider-initiated testing. Furthermore, data are only reported for reflexed CrAg testing, which is in line with local guidelines.^26^

In addition, the clinical aspects related to extending CrAg screening to a threshold of 200 cells/μL were not considered. The availability of public sector budgets to incorporate the additional costs into the national HIV and AIDS conditional grant was not considered.^27^ However, the Western Cape province has managed to extend screening using available public sector funding. The cost per result for CrAg testing is based on a national tender agreement with high test volumes. Higher costs may be reported for other settings with much lower test volumes. Furthermore, the cost to find one CrAg-positive case may also vary based on prevalence rates for a count between 100 cells/μL and 200 cells/μL, should this be broken up into multiple bins by absolute CD4 count.

## Conclusion

The study findings reveal that extending reflexed CrAg testing to a threshold of 200 cells/μL would double the AEC while finding fewer cases in the extended testing category eligible for antifungal treatment. A local prevalence study is required to provide the necessary evidence to conduct a cost-effectiveness analysis.
